# The impact of IGF-I, puberty and obesity on early retinopathy in children: a cross-sectional study

**DOI:** 10.1186/s13052-019-0650-x

**Published:** 2019-04-27

**Authors:** Carla Bizzarri, Stefania Pedicelli, Antonino Romanzo, Sarah Bocchini, Giorgia Bottaro, Stefano Cianfarani, Marco Cappa

**Affiliations:** 1 Unit of Endocrinology, “Bambino Gesù” Children’s Hospital, IRCCS, Piazza S. Onofrio 4, 00165 Rome, Italy; 2 Ophtalmology Unit, “Bambino Gesù” Children’s Hospital, IRCCS, Piazza S. Onofrio 4, 00165 Rome, Italy; 3 Unit of Diabetes, “Bambino Gesù” Children’s Hospital, IRCCS-Tor Vergata University, Piazza S. Onofrio 4, 00165 Rome, Italy; 4 Department of Women’s and Children’s Health, Karolinska Institutet, Stockholm, Sweden

**Keywords:** Obesity, Retinopathy, Puberty, IGF-I, Child

## Abstract

**Background:**

Childhood obesity has been correlated with coronary heart disease, but the correlation with microvascular disease remains unclear. The retinal microcirculation is affected early in the process of atherosclerosis and it offers the opportunity to indirectly study the effects of obesity on small brain vessels. Insulin-like growth factor 1 (IGF-I) is involved in angiogenesis and it has a crucial role in retinal vascularization.

**Methods:**

A single-centre cross-sectional study was performed in 268 children and adolescents (116 males; mean age 13.03 ± 1.9 years,) with overweight/obesity, in order to identify risk factors for early retinopathy.

**Results:**

Nine patients (3.3%) showed signs of retinopathy, defined as arteriovenous crossings and/or papilledema. Body mass index and fat mass, analysed by Dual X-ray Absorptiometry, were not different in patients with or without retinopathy. Patients with retinopathy were pubertal and showed higher waist circumference (107.78 ± 15.83 versus 99.46 ± 10.85 cm; *p:* 0.027), waist circumference/height ratio (0.66 ± 0.07 versus 0.62 ± 0.05; *p:* 0.04) and IGF-I SDS (0.03 ± 1.3 versus − 0.66 ± 0.9; *p:* 0.04). Multivariate analysis (after correction for sex, age, family history of type 2 diabetes mellitus, obesity, cardiovascular disease, hypertension and dyslipidaemia) showed that waist circumference/height ratio and IGF-I SDS were the only variables independently correlated with the presence of retinopathy.

**Conclusions:**

Retinal vascular changes may become evident as an early complication of overweight and obesity, even during childhood and adolescence. Relatively high levels of IGF-I during this phase may act as an additional risk factor for microvascular damage. The screening for retinopathy should be proposed to all children and adolescents with overweight/obesity.

## Background

In the last three decades, overweight and obesity have become a relevant health issue for a significant proportion of children and adolescents. Childhood obesity has been correlated with an increased risk of coronary heart disease and premature death in adulthood [1–3]. The obesity-associated impairments in the structure and function of large vessels have been definitely demonstrated; young children and adolescents with obesity show increased carotid artery intima-media thickness [4] and arterial stiffness [5].

On the contrary, the correlations between obesity, cardiovascular risk factors and early signs of microvascular disease in children remain to be elucidated. The retinal vasculature is easily analyzable by the use of a *fundus* camera. The retinal microcirculation is affected early in the process of atherosclerosis [6] and it offers the opportunity to study the effects of obesity on brain vessels. In adults, arteriolar narrowing and venular dilatation, as well as a reduced arteriolar-to-venular diameter (AVR) have been associated with an increased risk of hypertension and stroke, and with a higher cardiovascular mortality rate [6].

### Aims

A cohort of children and adolescents with overweight or obesity underwent clinical and metabolic assessment and *fundus oculi* examination, in order to identify clinical and hormonal risk factors for early retinopathy.

## Subjects and methods

### Subjects

A single-centre cross-sectional study was performed on a cohort of consecutive Caucasian Italian children and adolescents with overweight (BMI between + 1.75 and + 2 SDS) or obesity (BMI ≥ + 2 SDS). Children with overweight/obesity secondary to genetic syndromes or hormonal disorders were excluded. Table 1 summarizes the characteristics of the study population.

**Table 1:** Clinical and metabolic characteristics of the study group

Males/females (n°)	115/150
Prepubertal/Pubertal (n°)	43/222
Age (years)	13.04 ± 1.9
BMI SDS (Italy)	2.17 ± 0.5
Waist/height ratio	0.62 ± 0.05
Systolic blood pressure (mmHg)	120.5 ± 10.1
Diastolic blood pressure (mmHg)	67.08 ± 9.4
Fasting glucose (mg/dl)	85.53 ± 7.3
Fasting insulin (mU/L)	25.60 ± 17.2
Fasting C peptide (ng/ml)	2.07 ± 0.8
HOMA-IR	5.48 ± 3.9
QUICKI	0.31 ± 0.02
AUC glucose (mg/min/ml)	13,948.33 ± 2053
AUC insulin (microUI/min/ml)	17,154.3 ± 9057.2
AUC C peptide (ng/min/ml)	838.44 ± 307.8
HDL cholesterol (mg/dl)	46.78 ± 10.3
Triglycerides (mg/dl)	92.14 ± 52.3
C-reactive protein (mg/dl)	0.42 ± 0.7
IGF-I SDS	−0.64 ± 1.01

All patients underwent retinography, anthropometric examination, blood pressure measurement, basal biochemical assessment, oral glucose tolerance test (OGTT) and body composition analysis by Dual X-ray Absorptiometry (DXA). Family history of type 2 diabetes mellitus, obesity, cardiovascular diseases, hypertension or dyslipidaemia in first- and second-degree relatives was also investigated (positive/negative). The Institutional Review Board approved the study and all parents/patients gave their informed consent to participate.

A cohort of 268 patients (116 males; mean age 13.03 ± 1.9 years, range 6.6–18.8 years) was studied. The characteristics of the study population are summarized in Table 1.

### Clinical examination

All subjects underwent a general physical examination, performed by two paediatric endocrinologists (S.P. and S.B.). Weight was assessed by a digital scale, height by a Harpenden stadiometer. Body mass index (BMI) was calculated from the ratio of weight/height^2^ (kg/m^2^). BMI and height were expressed as SDS, using Italian reference data [7]. Puberty was assessed according to the Tanner’s criteria for female breast development [8] and according to a modified genital staging method based on the average volume of both testes in males [9].

Waist circumference was measured in a standing position, at the narrowest circumference between the lower costal margin and the iliac crest, with a tape measure to the nearest 0.5 cm. The waist to height ratio (W/Hr), which has recently been suggested as an index of abdominal adiposity in growing subjects, was assessed [10].

Systolic and diastolic blood pressure was measured after 30-min resting, while the subject was seated, and the average of the 3 measurements was used for the analysis.

### Laboratory tests

Basal blood samples were taken in the morning, after at least 8 h fasting. Triglycerides, total and HDL cholesterol were measured by an enzymatic colorimetric method. Creatinine, liver enzymes and blood count were assessed by automated methods.

Glucose, insulin and C-peptide were evaluated at 0, 30, 60, 90 and 120 min after oral administration of 1.75 g/kg (maximum 75 g) of glucose solution (OGTT). Plasma glucose was immediately measured by the glucose oxidase technique (Cobas Integra; Roche, Basel, Switzerland). Insulin and C peptide were measured by chemiluminescence on ADVIA Centaur analyzer. Intra- and interassay variability coefficients of insulin measurement were 4 and 5.5%, respectively. Intra- and inter-assay variability coefficients of C peptide measurement were 10 and 15%, respectively. To define insulin sensitivity, we calculated HOMA-IR (homeostasis model assessment-insulin resistance) as fasting plasma insulin (mU/L) x fasting plasma glucose (mmol/L)/22.5 and QUICKI (quantitative insulin sensitivity check index) as 1/[log10 fasting plasma insulin (mU/L) + log10 glucose (mg/dL)] [11].

The area under the curve (AUC) of glucose, insulin and C-peptide during OGTT were calculated using the trapezoid rule by adding the areas enclosed between each pair of consecutive observations (the shape of each area was considered as a trapezoid) [12].

Serum Insulin-like growth factor 1 (IGF-I) and IGF-binding protein 3 (IGFBP-3) levels were measured by chemiluminescence immunoassay (Immulite 2000-XPI). Mean intra- and interassay variability coefficients were 4.9 and 5% for IGF-I, and 4.1 and 8% for IGFBP-3, respectively. Age and sex related standard deviation scores (SDS) of IGF-I levels were considered for analysis.

### Retinography

The *fundus oculi* examination was performed by the same ophtalmologist (A.R.) using the TOPCON mod. TRC-NW200 NON-MYDRIATIC fundus camera. The photographic documentation was compared with Schepen’s indirect ophthalmoscopic exam.

The classification of Keith, Wagener and Barker [13] was used for the assessment of:

- Arterial light reflex, arterial narrowing, arterial sclerosis, arteriovenous crossings
- Retinal hemorrhages or retinal thrombosis
- Papilledema.

### Body composition analysis

Bone mass density (BMD) and body composition was evaluated using dual X-ray densitometry (DXA; Hologic Inc., Bedford, MA) with fan beam in the array mode. The same physician that examined the patient performed the scan. DXA scans were analyzed to generate measurements of lumbar spine BMD (L-BMD, g/cm^2^) and lumbar spine Z score (L-Z score), [14, 15]. Whole body scans were analyzed to generate whole body BMD (wbBMD, g/cm^2^) [16], as well as to evaluate body composition parameters: fat mass and lean mass, expressed as percentage of total body weight. Truncal fat was also evaluated and expressed as percentage of truncal weight.

### Statistical analysis

Data are presented as means ± standard deviation (SD) if not otherwise indicated. Unpaired two-tailed t-test was used to analyse the differences between patients with or without retinopathy. Proportions were compared by chi-squared test or Fisher’s exact test. A multivariable linear regression model was fitted in order to evaluate the independent effect of different covariates on retinopathy considered as outcome. A *p* value< 0.05 was considered significant. Statistical analysis was performed using SPSS (Version 17.0, SPSS, Inc., Chicago, IL).

## Results

A normal retinography pattern was found in 227 children (84.7%); 38 children (14.2%) showed congested retinal vessel and/or arteriovenous crossing and/or papilledema; different *fundus* alterations, as coloboma or choroidal nevus were found in 3 patients (1.1%).

Considering that coloboma and choroidal nevus could somehow prevent the adequate evaluation of the retinal vessels, the 3 patients presenting these features were excluded.

According to the assumption that the congestion of retinal vessels can be a transient and physiological condition during puberty [17], our study population was divided in:

- Group A: normal retinographic pattern or nonspecific retinal vessel alterations, as congested retinal vessel (*n* = 256; 96.6%);
- Group B: retinopathy, defined as arteriovenous crossing and/or papilledema (*n* = 9; 3.4%);

All patients of group B were pubertal. The assessment of metabolic syndrome (MetS) according to the IDF criteria [18] was completed in 256 patients. A full MetS was found in 29/256 patients (11.3%), 5 prepubertal patients and 24 pubertal patients. In Group B, 7/9 patients (77.8%) did not fulfil the IDF criteria for MetS; no patient showed a fasting glucose ≥100 mg/dl, one patient showed a systolic blood pressure ≥ 130 mmHg, and no patient showed diastolic blood pressure ≥ 85 mmHg. Family history of hypertension was positive in 70% percent of group B children, and 56.2% of group A children (*p*: 0.36).

Patients with retinopathy showed higher values of waist circumference and waist circumference/height ratio (Table 2). No further differences in hormonal and metabolic parameters were found, with the exception of significantly higher IGF-I SDS (Group A: − 0.66 ± 0.9 versus Group B: 0.03 ± 1.3; *p*: 0.04). The severity of excess weight and body composition parameters at DXA were not significantly different in the 2 groups (Tables 2 and 3).

**Table 2:** Clinical, hormonal and metabolic characteristics according to retinopathy

	Group A (normal)	Group B (retinopathy)	*P*
Males/females (n°)	110/146	5/4	0.56
Prepubertal/Pubertal (n°)	43/213	0/9	0.36
Age (years)	13.04 ± 1.9	12.9 ± 1.02	0.81
BMI SDS (Italy)	2.17 ± 0.5	2.39 ± 0.7	0.23
Waist circumference (cm)	99.46 ± 10.85	107.78 ± 15.83	0.027
Waist/height ratio	0.62 ± 0.05	0.66 ± 0.07	0.04
Systolic blood pressure (mmHg)	120.5 ± 10.1	121.8 ± 10.8	0.72
Diastolic blood pressure (mmHg)	67.1 ± 9.4	67.7 ± 10.9	0.83
Fasting glucose (mg/dl)	85.5 ± 7.3	86.3 ± 7.2	0.73
Fasting insulin (mU/L)	25.67 ± 17.4	23.2 ± 10.7	0.69
Fasting C peptide (ng/ml)	2.07 ± 0.7	2.01 ± 0.75	0.84
HOMA-IR	5.5 ± 3.9	5.05 ± 2.6	0.43
QUICKI	0.31 ± 0.02	0.30 ± 0.01	0.58
AUC glucose (mg/min/ml)	13,950,12 ± 2070.7	13,901 ± 1616,8	0.94
AUC insulin (microUI/min/ml)	17,271.05 ± 9064.23	14,993.47 ± 9471.56	0.55
AUC C peptide (ng/min/ml)	838.3 ± 306.8	841.98 ± 352.7	0.97
HDL cholesterol (mg/dl)	46.66 ± 10.1	49.7 ± 12.8	0.37
Triglycerides (mg/dl)	92.26 ± 52.5	88.55 ± 50.3	0.83
C-reactive protein (mg/dl)	0.41 ± 0.7	0.33 ± 0.3	0.72
IGF-I SDS	−0.66 ± 0.9	0.03 ± 1.3	0.04

**Table 3:** Body composition analysis according to retinopathy

	Group A (normal)	Group B (retinopathy)	*P*
L-BMD (g/cm^2^)	0.87 ± 1.77	0.86 ± 0.1	0.92
L-Z score	0.63 ± 1.1	0.72 ± 1.05	0.83
wbBMD (g/cm^2^)	1.03 ± 0.1	1.07 ± 0.08	0.43
Total fat (%)	41 ± 4.1	40.2 ± 4.1	0.65
Truncal fat (%)	40.51 ± 5.2	39.9 ± 4.1	0.74
Total lean (%)	57.57 ± 6.1	57.3 ± 3.5	0.9

Multivariate analysis, after correction for covariates (sex, age, family history of type 2 diabetes mellitus, obesity, cardiovascular diseases, hypertension and dyslipidemia), showed that waist circumference/height ratio and IGF-I SDS were the only factors independently correlated with retinopathy. Table 4 shows the results of multivariate analysis in its most parsimonious form, which retains just the covariates significantly associated with the outcome.

**Table 4:** Multivariable analysis of factors independently associated with retinopathy

Independent variable	Beta coefficient	95% Confidence interval	*P value*
Waist/height ratio	0.148	from 0.074 to 0.939	0.022
IGF-I SDS	0.146	from 0.004 to 0.051	0.024

R^2^: 0.038; adjusted R^2^: 0.030

## Discussion

Childhood obesity has been shown to be a major risk factor for the development of cardiovascular disease and a predictor of premature death in adulthood [1–3]. It has been suggested that arterial hypertension may originate early in life and changes of microvascular structure represent the underlying mechanism leading to the development of hypertension [19]. This is the first study to demonstrate that early signs of retinopathy with arteriovenous crossings are already evident during puberty. These changes seem to be positively associated with IGF-I levels and they probably represent an early complication of overweight and obesity, even before the onset of arterial hypertension and other components of the metabolic syndrome.

A few studies had previously examined the effects of body adiposity, blood pressure, and metabolic characteristics on retinal vessels in children.

Mitchell and coll. [20] in 2007 examined the relationship between blood pressure levels and retinal vascular calibre in 2 different population-based cohorts of children aged 6–8 years in Australia (1572 children) and Singapore (380 children). Participants underwent digital retinal photographs and measurement of retinal arteriolar and venular calibres. Children with higher blood pressure had significantly narrower retinal arterioles than those with lower blood pressure. After correction for age, sex, race, BMI, refraction, and birth parameters, each 10-mmHg increase in systolic blood pressure was associated with narrowing of the retinal arterioles by 2.08 μm in Australian children and 1.43 μm in Singapore children. Retinal venules were not affected by blood pressure.

Hanssen and coll. [21] in 2012 examined a group of 578 children (age 11.1 ± 0.6 years), including 128 children with overweight or obesity (22.2%). Physical activity was evaluated by a questionnaire. Retinal microvascular diameters were assessed as central retinal arteriolar and venular equivalents (CRAE, CRVE), and AVR with a non-mydriatic vessel analyser using a computer-based program. Venular diameters were wider in children with obesity compared to children with normal weight, while arteriolar diameters were not significantly different. Children with overweight/obesity had lower AVR. Wider venular diameters were independently associated with higher BMI and higher C-reactive protein levels. Blood pressure was associated with retinal vessel constriction. Sedentary life-style and BMI were independently associated with a reduced AVR.

Kurniawan and coll. [22] in 2014 reported the results of a longitudinal study in 421 healthy children. Enrolled children (aged 7–9 years) were first examined in 2001 and returned for follow-up in 2006. Retinal vessel calibers were measured using a computer-based program and summarized as central retinal arteriolar equivalent (CRAE) and central retinal venular equivalent (CRVE). In the 5-year interval between baseline and follow up visits, mean weight, height, and BMI significantly increased. Mean CRVE increased by 3.4 μm, but mean CRAE did not change significantly. On longitudinal analysis, increasing BMI was associated with increasing CRVE. Considering that both widened retinal venular calibre and greater BMI are associated with cardiovascular risk in adults, the authors supposed that the progressive venular widening could represent an early sign of the detrimental effect of obesity on retinal vessels.

Gishti and coll. [23] in 2015 performed a population-based cohort study among a wide cohort of children (median age 6.0 years). Children with obesity showed narrower retinal arteriolar calibre than normal weight children, but venular calibres were not different. Higher BMI and total body fat mass, but not fat mass distribution, were associated with narrower retinal arteriolar calibre. Lipid and insulin levels were not associated with retinal vessel calibre. Higher C-reactive protein was associated only with wider retinal venular calibre, and BMI did not influence this association.

Liccardo and coll. [24] in 2015 analysed 54 children with BMI > 85°centile and biopsy proven non-alcoholic fatty liver disease. Retinal vascular calibre was quantified from digital retinal images using computer-based programs. Retinal vessel tortuosity was evaluated simultaneously for both eyes and reported as the average evaluation of the right and left eye. The grading levels for retinal arterial tortuosity were category 1 (normal): predominantly straight arteries in all four quadrants; category 2: wavy, mild-to-moderate tortuosity, with one or two inflections of at least one major artery in one to three quadrants; category 3: prominent tortuosity with two or more inflections of at least one artery in all four quadrants. Twenty-nine patients (53%) showed retinopathy: 21 patients with category 2 retinopathy and 8 patients with category 3 retinopathy. Patients with retinopathy showed significantly higher levels of triglycerides, basal insulin, and HOMA-IR. The category of retinopathy was positively correlated with the severity of non-alcoholic fatty liver disease.

To our knowledge, this is the first study to suggest an association between retinal vascular abnormalities and puberty in children with overweight/obesity. The small number of subjects with retinopathy clearly limits the power of analysis. The prepubertal/pubertal ratio was not statistically different in children with or without retinopathy, and chronological age was similar in the two groups. Nevertheless, it seems noteworthy that all children with retinopathy were pubertal. This finding suggests a significant role of the physiological insulin-resistance that characterizes puberty in determining the onset of the chronic microvascular complications of obesity. The complex hormonal storm occurring during puberty in both sexes, is characterized by increased plasma levels of growth hormone, IGF-I and gonadal steroids and could contribute to the beginning of the microvascular damage, as already reported for diabetic retinopathy [25]. IGF-I levels in obese children have been reported to be high [26] or normal [27], although growth hormone secretion is generally low [26, 27]. Interestingly, overall IGF-I levels (expressed as age and sex related SDS), were not elevated, but significantly higher in subjects with retinopathy. Multivariate analysis confirmed a positive relationship between IGF-I level and retinopathy, which was independent of age, sex, and family history of type 2 diabetes mellitus, obesity, cardiovascular diseases, hypertension and dyslipidemia. Our patients had not undergone 24 h-blood pressure monitoring, thus it is difficult to completely rule out the impact of any underlying hypertension. Moreover, due to the cross-sectional design of the study we cannot exclude that early retinopathy in pubertal children may be transient, similarly to what described in adult hypertensive retinopathy that can regress after an adequate anti-hypertensive treatment [13]. It is well known that IGF-I is involved in angiogenesis and it has a crucial role in normal and pathological vascularization of the retina [28–30]. Vitreal IGF-I levels were found to be increased in diabetic patients and IGF-I seems to be involved in retinal neovascularization, which is the primary determinant of prematurity-related retinopathy and proliferative diabetic retinopathy [28]. IGF-I and IGF-I receptors have been detected in retinal endothelial cells, lens epithelial cells and retinal pigment epithelium [29, 30].

The severity of obesity expressed as BMI SDS and obesity-related comorbidities (such as insulin resistance, increased blood pressure, or an adverse lipid profile) did not correlate with retinopathy. However, multivariate analysis showed that the presence of retinopathy was independently related to a higher waist/height ratio, while it was not influenced by a positive family history of type 2 diabetes mellitus, obesity, cardiovascular diseases, hypertension and dyslipidemia. The waist/height ratio is confirmed to be more effective than waist circumference as an index of abdominal adiposity in growing subjects, as it allows an early assessment of adipose tissue re-distribution and central fat accumulation. This simple method seems to be even more reliable than the assessment of body composition by DXA. An increased waist/height ratio seems to be associated with insulin resistance and higher metabolic and cardiovascular risk, even before the development of overt obesity and metabolic syndrome [10, 31].

## Conclusions

The retinal microcirculation is affected early in the process of atherosclerosis and it offers the opportunity to study the effects of obesity on small brain vessels. Our preliminary results suggest that early retinal vascular changes may become evident even during childhood and adolescence, as an early complication of overweight and obesity. IGF-I levels during this phase appear to influence the risk of microvascular damage. IGF-I levels seem to be relatively higher in subjects with retinopathy, while low IGF-I levels could have a protective role.

A longitudinal study is definitely needed to investigate the clinical significance and the long-term evolution of these early retinal lesions.

The retinal vessels are easily analyzable by the use of a *fundus* camera, without any significant distress for patients, even in childhood. The screening for retinopathy by retinal photographs should be proposed at diagnosis and during follow-up to all children and adolescents with overweight and obesity.

## Abbreviations

AUC: Area under the curve
BMD: Bone mass density
BMI: Body mass index
DXA: Dual X-ray densitometry
HOMA-IR: Homeostasis model assessment-insulin resistance
L-BMD: Lumbar spine BMD
OGTT: Oral glucose tolerance test
QUICKI: Quantitative insulin sensitivity check index
SD: Standard deviation
SDS: Standard deviation score
wbBMD: Whole body BMD
